# On Yellow Bark

**Published:** 1844-03-23

**Authors:** 


### ON YELLOW BARK.

From a variety of experiments performed by Mr. Battley, the following conclusions are drawn.

1. Bark will yield to *cold distilled water* all its constituents except starch and woody fibre, some earthy salts, and a small portion of tannin and quinine, which can only be separated from the tissue by means of an acid.

2. 28 lbs. of yellow bark will yield from 5 to 6 lbs. of concentrated liquor, sp. gr. 1200, containing about 10 oz. quinine; the aroma and the greater part of the tannin and iron, and the peculiar acid of bark, of which only a small portion is lost, forming an inert salt with lime.

3. To form this liquor it is only necessary to subpulverise the bark, and macerate it from four to six hours in twice its weight of cold distilled water, repeating the process twice or at most thrice; to concentrate the infusions over a water-butt to sp. gr. 1200, and allow the liquor to deposit the gummy matter and so much of the tannin as it cannot retain in solution.

To separate the gum that may still remain in the liquor, and to prevent any decomposition, proof spirit is added to it, until the sp. gr. of the liquor is reduced to 1100. The quinine still remaining in the bark, may, if it be thought desirable, be separated by acetic acid, and precipitated from its solution by ammonia; and being redissolved in a small quantity of dilute acetic acid, may be diffused in the liquor.

The advantages of this medicine are—

1. That it contains not one, but all the active principles of yellow bark.

2. That the greater part of the quinine is preserved *in its natural state* in combination with the peculiar acid of bark, in which it is more soluble than in sulphuric acid.

3. That the active principles *have undergone no change*, either in the exposure to too great heat, as in the decoction, &c. or by being brought into too close contiguity, as in the extract, in which secondary formations take place to so great an extent that water is incapable of redissolving it.'

4. That, containing no starch and little gum, it will remain unaltered for a great length of time.

5. That the quantity of spirit contained in a dose is too small to be objectionable.

6. That it is a convenient, agreeable, and elegant medicine, miscible with wine or water in any proportions.

The above is a *pharmaceutical* analysis of yellow bark; Mr. Battley hopes soon to prosent a *chemical* analysis, as well as a similar set of experiments on the cinchona lancifolia.—*Medico-Chirurgical Review, from Medical Gazette.*

